# Attraction of Insects to Ornamental Lighting Used on Cultural Heritage Buildings: A Case Study in an Urban Area

**DOI:** 10.3390/insects13121153

**Published:** 2022-12-14

**Authors:** Anxo Méndez, Luis Martín, Justo Arines, Rafael Carballeira, Patricia Sanmartín

**Affiliations:** 1 GEMAP (GI-1243), Departamento de Edafoloxía e Química Agrícola, Facultade de Farmacia, Universidade de Santiago de Compostela, 15782 Santiago de Compostela, Spain; 2 Departamento de Física Aplicada, Facultade de Óptica e Optometría, Universidade de Santiago de Compostela, 15782 Santiago de Compostela, Spain; 3 iMATUS (Instituto de Materiais), Universidade de Santiago de Compostela, 15782 Santiago de Compostela, Spain; 4 Centro de Investigacións Científicas Avanzadas (CICA), Facultade de Ciencias, Universidade da Coruña, 15008 A Coruña, Spain; 5 CRETUS, Universidade de Santiago de Compostela, 15782 Santiago de Compostela, Spain

**Keywords:** artificial light at night (ALAN), biodiversity, flight-to-light behaviour, insect decline, light-emitting diode (LED), pilot study, public lighting, Santiago de Compostela

## Abstract

**Simple Summary:**

The increasing use of ornamental illumination has an impact on nocturnal insect communities in urban areas. The objective of this study is to verify if the selection of specific wavelengths reduces the attraction of insects towards ornamental lighting. We compared the number and diversity of insects captured onto grey sticky traps of an unilluminated area with two light sources: a metal halide lamp and a more efficient, environmentally sound prototype lamp (CromaLux) comprising a combination of green and amber LEDs. By limiting the light emitted to amber and green, the CromaLux lamps considerably reduced the attraction to the light, with similar numbers captured as in the unilluminated area, while the metal halide lamp attracted a greater number and diversity of insects.

**Abstract:**

Artificial light at night (ALAN) reduces insect populations by altering their movements, foraging, reproduction, and predation. Although ALAN is mainly associated with streetlights and road networks, the ornamental illumination of monuments is making an increasing (but not well-studied) contribution. We compared insect attraction to two different types of light sources: a metal halide lamp (a type currently used to illuminate monuments) and an environmentally sound prototype lamp (CromaLux) comprising a combination of green and amber LEDs. The experiment was performed within the pilot CromaLux project in Santiago de Compostela (NW Spain). The abundance and diversity of the insects captured between June and October 2021 in the areas surrounding both light sources and in an unlit area were compared. By limiting the light emitted to amber and green, the CromaLux lamps reduced the number and diversity of insects, morphospecies, and orders attracted to the light, with similar numbers captured as in the unilluminated area, while a greater diversity of insects was captured beside the metal halide lamp. This effect has been demonstrated for almost all insect orders trapped, especially in Diptera, Lepidoptera, Coleoptera, Hemiptera, and Hymenoptera. On the contrary, Psocoptera showed a similar attraction to the CromaLux and metal halide lamps, a phenomenon whose causes deserve further investigation. As expected, Diptera were the most diverse and abundant insects in all samples, but the abundance of Lepidoptera was unexpectedly low (4%), which is in line with the worldwide evidence of the progressive decline of populations of this group. The study findings provide evidence that selecting specific wavelengths for ornamental lighting reduces the attraction of insects while maintaining adequate illumination of monuments for aesthetic purposes, resulting in a lower environmental impact on nocturnal insects. This study provides reference data for developing principles of good practices leading to possible regulatory and legal solutions and the incorporation of specific measures for artificial lighting of monuments and urban structures.

## 1. Introduction

Night-time illumination of architectural objects has been common practice for centuries [1], enhancing historical or important constructions and distinguishing them from other buildings while also strengthening the significance of the cultural identity of cities [2]. The shift towards more sustainable lighting technologies with the development of light-emitting diodes (LEDs) has reduced the energy consumption and economic cost of urban illumination. However, LEDs are still problematical, as the light emitted glows in the night sky, reducing the level of darkness. In fact, 88% of the land surface in Europe experiences nightglow [3], disrupting the natural day–night cycles of many organisms [4,5,6] and luring insects and birds to their deaths [7]. Nightglow is increasing globally at a rate of 2% per year [8]; in 2014, the contribution made by the ornamental lighting of cultural heritage objects to the nightglow was between 5% and 20% [9], rising to 21% in Spain in 2018 [10].

Artificial light at night (ALAN) has a serious impact on diverse organisms, many of which (around 30% of all vertebrates and more than 60% of all invertebrates) display nocturnal behaviour, with, e.g., reproduction and feeding taking place during night hours [3]. Insects are declining rapidly around the world [11], and the widespread adoption of ALAN presents a growing threat to the biodiversity of insects in general and of nocturnal insects in particular. Thus, ALAN causes (i) the mortality of nocturnal insects due to direct collisions with the lamps, (ii) exhaustion from continuous circling around the light, (iii) temporal disorientation, desynchronization of natural biorhythms, and (iv) spatial disorientation, which interferes with navigation in nocturnal landscapes [12,13,14,15], along with indirect effects such as enhancement of predation by bats or other predators [16] and parasitism of moth larvae [17,18].

Furthermore, the frequent replacement of ornamental public lighting sources modifies the flight-to-light behaviour of insects [15], accustomed since the first half of the last century to mercury vapour lamps and fluorescent lights [19], which emit UV radiation that is extremely attractive to many nocturnal insects [13,14,20]. Still used today, these light sources coexist with the high- and low-pressure sodium lamps that became popular in the 1950s [21] and with the more economical metal halide lamps [22]. These types of lights, which are increasingly being replaced with LED lamps, enable greater control over the dominant wavelength and correlated colour temperature (CCT), i.e., whether a white light is relatively more blue or yellow–amber in terms of shades of white; in other words, its colour appearance expressed in Kelvin (K).

Although extensive research has addressed the effects of night-time illumination on insects, the specific effects and contributions of ornamental illumination of monuments are little known, with only a few studies focusing on wooded areas [23] and, to the best of our knowledge, no studies in urban areas.

The present study formed part of a larger research project entitled “CromaLux: Development of a lighting system with artificial intelligence support for the control of biological colonization in elements of high heritage value (2020–2022)” (The CromaLux project. Available online: http://cromalux.santiagodecompostela.gal/en, accessed on 13 December 2022, Figure 1), conducted in the city of Santiago de Compostela (NW Spain). The aim of the present study was to compare the numbers and diversity of insects attracted to an innovative LED lamp (CromaLux, currently under trial) emitting amber and green light, those attracted to a metal halide lamp (positive control), and those attracted to an unilluminated area (negative control). We tested two main hypotheses: (i) the novel LED lamps will attract significantly fewer insects than the metal halide lamps, with numbers similar to those in the unilluminated area, and (ii) the composition of the insect community will be significantly less affected by the LED lamps than by the metal halide lamps.

## 2. Materials and Methods

### 2.1. Study Location and Sampling Set-Up

The study was conducted in the historical centre of Santiago de Compostela (Figure 2a), a UNESCO World Heritage city in northwestern Spain. The experiments were conducted in an inner courtyard of an area of approximately 245 m^2^ (the old prison yard of the local police building, located behind the city council building, Pazo de Raxoi: UTM 537047 X, 4747615 Y, Datum ETRS89; elevation, 250 m a.s.l.) (Figure 2b). Sticky coloured boards were used to trap the insects, as in previous studies [9,24,25]. The boards, of a length of 24.5 cm and a width of 42.7 cm, and each marked with 100 rectangles of a length of 4.0 cm and a width of 2.3 cm, were obtained from PestWest (Sarasota, FL, USA). Preliminary tests were carried out with sticky boards of different colours (black, yellow, and grey) to check the light reflectance (with a spectroradiometer SilverNova, StellarNet, Inc., Tampa, FL, USA) and suitability for the identification of insects. The black board was rejected because it was very difficult to identify the insects due to the low contrast. The yellow board was rejected due to the yellowish colour of the reflected spectrum. Finally, the grey board was selected for the experiments, as it provided a good contrast, enabling the insects to be observed against the background. Furthermore, we confirmed with a spectroradiometer (SilverNova, Stellarnet, Inc.) that the grey board did not change significantly the reflected spectra. The sticky board traps (PestWest, Sarasota, FL, USA) were placed under the CromaLux lamp (under trial) and the metal halide lamp (positive control) at a height of three metres (Figure 2c). The sticky board trap used in the unlit area (negative control) was placed on a wall without any artificial illumination at the same height and orientation as the other two traps (Figure 2d). The sticky traps were removed and replaced after 5–16 days, depending on the number of insects trapped on the board, under the metal halide lamp (helping insect identification by avoiding saturation) and also on the probability of rain, which could damage the boards. A total of fifteen replacements were made (forty-five sticky traps in total), and these were grouped into five similar time periods for data analysis (Table 1).

### 2.2. Artificial Lighting and Environmental Conditions

Both types of lamps were switched on for five hours (between 22:00 h and 03:00 h) every night during the study period. The spectra of the lamps were measured with a radiometrically calibrated spectroradiometer (SilverNova, Stellarnet, Inc.) with a spectral resolution of 5 nm. The temperature of the sticky board traps while the lamps were switched on was monitored with HOBO MX2202 data logger (Onset, Bourne, OR, USA). The humidity in the inner courtyard was monitored with a HOBO MX2302A data logger (Onset, Bourne, OR, USA). These data were completed with data on precipitation, daylight hours, and average temperature and relative humidity throughout the study period from the web-based repository www.meteogalicia.gal (the Galician meteorological service) (Table 1).

### 2.3. Insect Collection and Identification

Insects were collected during the warmest time of year in the study location, between 1 June and 20 October 2021 (Table 1), covering the main activity period (caused by the mild temperatures and no high rainfall) of flying insects in the region [26,27,28]. After collection, (see the Section 2.1), the sticky boards were transported to the laboratory in a box that kept them cool and dry (conditions that were maintained for storage throughout the study). In the laboratory, each sticky board was photographed (with a Canon EOS 100D camera), and the number of insects on each board was counted. The insects were examined under a stereomicroscope (Olympus SZX7, Olympus, Hamburg, Germany) and photographed in greater detail (with an Olympus C180 digital camera, Olympus, Hamburg, Germany). All specimens or individuals were sorted, identified, and classified by a trained entomologist to recognizable taxonomic units (or morphospecies) and order level, and, when possible, to suborder, family, and genera/species levels. Taxonomic keys and manuals used included [29] for the general study of Iberian arthropods, [30] for Trichoptera, [31] for Diptera, [32], for other taxa, and the web-based repository Fauna Ibérica project (Available online: www.faunaiberica.es/, accessed on 13 December 2022) for verification of the nomenclature.

### 2.4. Biodiversity and Statistical Analysis

The diversity of morphospecies for each lighting condition was evaluated using the Shannon–Wiener index, a popular metric used in ecology, that summarise the diversity of a community quantifying the number of individuals and their relative abundance [33]. Differences between lighting conditions were determined using Hutcheson’s *t*-test [34] and were considered statistically significant at *p* > 0.05.

The data on the insects captured on the sticky board traps were analysed using the nonparametric Kruskal–Wallis test (K), as the assumptions of normal distribution were not fulfilled (Shapiro–Wilk test; *p* > 0.05). A post hoc Conover–Iman test © was used for multiple pairwise comparisons (*p* < 0.05) [35,36]. The temperature data from the loggers fitted to the sticky boards were also analysed in the same way. The data on the morpho-species, with specimens in the three lighting conditions, or with more than 10 specimens in one of the conditions, were previously selected for the statistical analysis, and a logarithmic transformation was applied. The data analysis was conducted using R statistical software (v. 4.0.2., R Core Team 2020, Vienna, Austria) [37].

## 3. Results

The optical spectra of both illumination systems under study are shown in Figure 3. The prototype LED lamp (CromaLux) yields a bimodal spectrum with two peaks, one at 528 nm, with full width at half maximum (FWHM) of 14 nm, and a main peak at 593 nm, with FWHM of 7 nm, producing a correlated colour temperature (CCT) of 3000 K. The spectrum of the metal halide lamp (positive control) falls in the visible range, with five main peaks centred at 435 nm, 508 nm, 546 nm, 578 nm, and 589 nm, with other important peaks at 473 nm, 569 nm, and 624 nm. The spectrum includes large amount of ultraviolet-A (UVA) light in the range 350–400 nm, with a peak at 365 nm. The lamp provided a CCT of 4668 K.

The temperature did not differ significantly between the sticky board traps beside the metal halide (12.25 ± 2.42 °C) and CromaLux (12.13 ± 2.53 °C) lamps, while the temperature of the traps in the unilluminated area was significantly higher (16.97 ± 1.75 °C). These results may lead to think that the negative control should have been placed at a height of three metres in the centre of the inner courtyard, as with its two counterparts, instead of on the wall (Figure 2). However, there was no unlit areas except for on the walls and it was considered that placing the trap on the wall would overestimate the impact results, never otherwise, because the temperature there is higher. The relative humidity at the study site was 80.92 ± 0.43%, which is consistent with the data extracted from the web-based repository, ranging sequentially from 78.95 ± 7.91% at the beginning to 87.88 ± 6.31% at the end of the experiment (Table 1). The average air temperature peaked in August, at 18.30 ± 1.54 °C, and was minimal in October, at 14.72 ± 1.26 °C. Rainfall levels were only 0.58 ± 1.38 L m^−2^ in July and 0.44 ± 1.30 L m^−2^ in August, but were higher in October, at 4.27 ± 8.58 L m^−2^. Finally, sunshine duration decreased, as expected, from summer to autumn.

A total of 1804 specimens or individuals and 160 insect morphospecies were captured on the sticky boards during the study period (Table 2, Figure 4). Nine insect orders were identified across all samples: Diptera represented 47% of the insects trapped (842 specimens and 65 morphospecies); Hemiptera represented 25% (457 specimens and 28 morphospecies); Psocoptera represented 15% (268 specimens and 8 morphospecies); Coleoptera represented 5.5% (100 specimens and 14 morphospecies); Lepidoptera represented 4% (74 specimens and 24 morphospecies); Hymenoptera represented 2.6% (47 specimens and 14 morphospecies); Neuroptera represented 0.3% (6 specimens and 1 morphospecies); and Trichoptera and Thysanoptera represented, respectively, 0.3% and 0.2% (6 and 4 specimens and 3 morphospecies in each order). It is summarized in Table 2. The order level, and when it was possible, the suborder, family, and genera/species levels of the insects trapped are also summarized in Table S1. The most abundant suborders of Diptera were Nematocera (24 morphospecies) and Schizophora (7 morphospecies), including the taxa Chironomidae, Psychodidae, Tipuloidea, and Muscidae. Hemiptera specimens comprised the suborders Cicadomorpha (18 morphospecies, mainly belonging to the family Cicadellidae, e.g., *Cicadella viridis* (Linnaeus, 1758) at the species level), Heteroptera (5 morphospecies), Sternorrhyncha (4 morphospecies), and Aphidodea (1 morphospecies). All Psocoptera specimens caught belonged to the suborder Psocomorpha. Polyphaga (12 morphospecies) was the most abundant suborder of Coleoptera, including species of Bostrichidae, Chrysomelidae, Cryptophagidae, Latridiidae, Nitidulidae, Ptiliidae, Silvanidae, Staphylinidae, and Tenebrionidae families, and genera such as *Cybocephalus* and *Schistocerus*. Within the order Coleoptera, specimens of the family Carabidae (suborder Adephaga) were also found. Lepidoptera species with nocturnal or crepuscular habits, mainly belonging to the family Noctuidae, such as *Lacanobia oleracea* (Linnaeus, 1758) and *Noctua pronuba* (Linnaeus, 1758), were captured. Trapped Hymenoptera specimens mainly included the suborders Apocrita (Chrysididae, Formicidae, and Ichneumonidae families) and *Symphyta* (Cephidae family). The order Neuroptera included a single species, i.e., *Chrysoperla carnea* (Stephens, 1836), which belongs to the family Chrysopidae, suborder Hemerobiiformia. Species of *Trichoptera*, i.e., *Lepidostoma basale* (Kolenati, 1848), which belong to the family Lepidostomatidae, suborder Integripalpia, and *Wormaldia* (McLachlan, 1865), which belongs to the family Philopotamidae, were also found.

The number of insects trapped differed significantly between the three lighting conditions (CromaLux lamp, metal halide lamp, and no illumination), according to the results of the Kruskal–Wallis test (K = 66.5; *p*-value < 0.0001) and the post hoc Conover–Iman test for pairwise comparisons (no illumination vs. metal halide lamp, C = 58.050, *p*-value < 0.0001; no illumination vs. CromaLux lamp, C = 12.263, *p*-value = 0.016; metal halide lamp vs. CromaLux lamp, C = 45.788, *p*-value < 0.0001), with higher values for the metal halide lamp (1421 insects) than for the CromaLux lamp (260 insects) and no illumination (114 insects) (Figure 5). Relative to the number of insects captured in the unilluminated control area, roughly twelve times more were attracted to the metal halide lamp and roughly two times more to the CromaLux lamp.

Figure 6 shows the distribution of the number of individuals and the number of morphospecies per insect order under the three lighting conditions. A total of 1421 individuals and 145 morphospecies were trapped beside the metal halide lamp. Values were significantly higher than those obtained for the CromaLux lamp and the unilluminated area, where respectively 260 and 114 individuals, and 62 and 40 morphospecies were caught. The number of individuals and morphospecies captured beside the CromaLux lamp was lower than in the positive control for all nine orders, except Psocoptera and Thysanoptera. Members of the orders Neuroptera and *Trichoptera* were absent from the traps beside the CromaLux lamps and in the unilluminated area, while Thysanoptera was absent from the traps beside the metal halide lamp. Although more morphospecies were captured beside the CromaLux lamp than beside the metal halide lamp, Lepidoptera morphospecies were only found on the latter. Members of the order Psocoptera were caught in equal numbers on the sticky traps beside the metal halide lamp (133) and the CromaLux lamp (128), while for the other orders greater numbers of insects were captured beside the metal halide lamp.

The changes in the number of individuals and morphospecies of each insect order throughout the experiment (Table 1) are shown in Figure 7. Except for some individuals of the order Psocoptera on the CromaLux, the orders Diptera and Hemiptera were the most abundant (by more than 60%) in all samples. Members of the order Hymenoptera were only present during the summer months. Insect abundance (Figure 7A) in the unilluminated area and beside the CromaLux lamp increased until August and then decreased sharply, the former in September and the latter in October. The number of members of Psocoptera captured on the traps beside the CromaLux lamp increased in the last three sample collections, representing just over half of the individuals captured in August, September, and October.

The number of specimens captured beside the metal halide lamp remained high and constant until September and then decreased sharply in October. Figure 7B shows the changes in morphospecies over time. The total number of morphospecies followed a similar pattern for the metal halide and CromaLux lamps (though not the same scale, ranging between 70 and 50 and 30 morphospecies number in the former and between 23 and 17 and 10 morphospecies number in the latter) with peaks in July and September, somewhat lower values in June, and to a lesser extent in August, and significant lower numbers in October. However, the number of morphospecies captured in the unilluminated area increased significantly between June and July, and then decreased gradually until October. The global variation (all data) in morphospecies diversity (Shannon–Wiener index; data used for the calculation appear in Table S2) was 2.80 for the unilluminated area, 4.07 for the metal halide lamp, and 2.95 for the CromaLux lamp. The monthly variation in the index (Figure 7B) ranged from 1.54 (October) to 2.59 (July) for the negative control, from 2.93 (October) to 3.86 (August) for the metal halide lamp, and from 1.74 (October) to 2.63 (June) for the CromaLux lamp. The Hutcheson t-test did not reveal any differences in the lighting conditions between the unilluminated area and the area lit by the CromaLux lamp (*p*-value = 0.401) but did reveal significant differences between the area lit by the metal halide lamp and the unilluminated area (*p*-value < 0.001) and the metal halide and CromaLux lamps (*p* < 0.001).

## 4. Discussion

The general use of artificial light at night (ALAN) may affect human perception [2], human health [39], and biodiversity [11]. It will also impact nocturnal insects, most of which display positive phototaxis at night and a large proportion of which have trichromatic vision, with receptor sensitivities in the ultraviolet, blue, and green spectral range [40]. Spectral composition is thus a key factor in insect attraction [18], and lights with peak wavelengths in blue and ultraviolet regions have been widely used to trap nocturnal insects in taxonomic and ecological research [41,42] and in pest control [24]. However, regarding ALAN, the focus is centred on streetlights and road networks, and scant attention has been given to ornamental or artistic illumination despite the increasing trend for cities to install external lighting systems on historical buildings and monuments [43,44].

**Figure 1:**
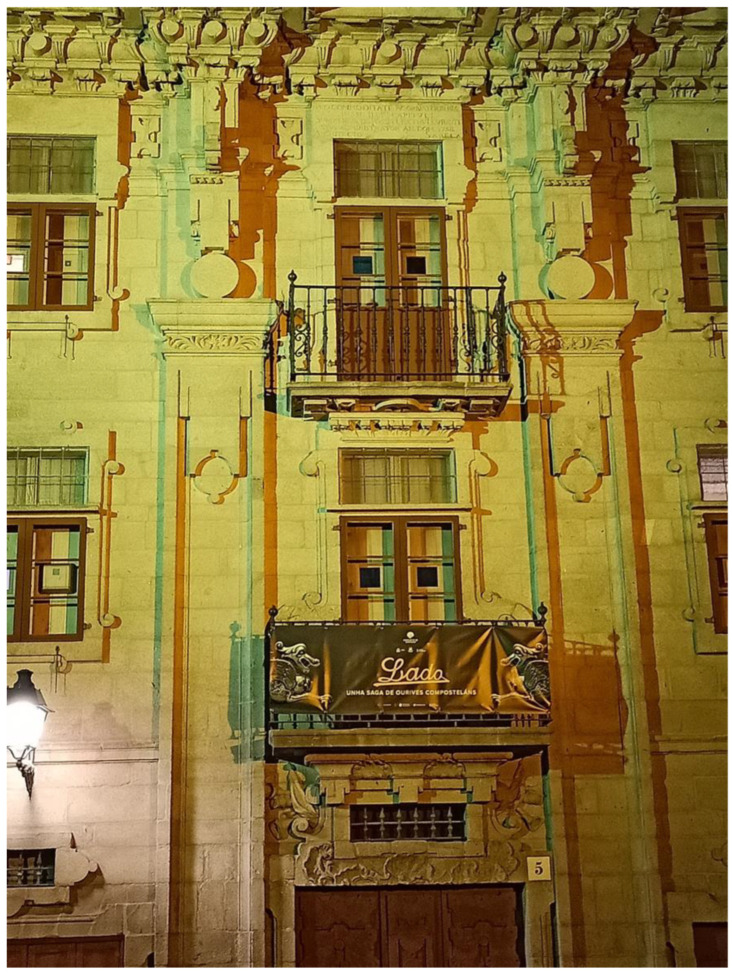
The baroque facade of the Casa do Cabildo house, built in 1758, illuminated by the CromaLux lighting.

**Figure 2:**
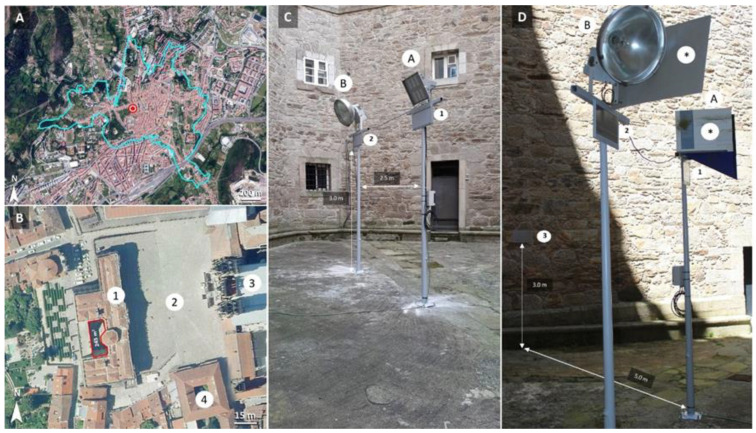
Experimental layout. (**A**) Overview of the historical centre of Santiago de Compostela (outlined in blue) and the location of the study site (red point). (**B**) Study site, inner courtyard (outlined in red), 1: Pazo de Raxoi (city council buildings), 2: Praza do Obradoiro (Obradoiro Square), 3: Cathedral of Santiago de Compostela, 4: Colexio de San Xerome (a university building). (**C**,**D**) Experimental set-up details in the inner courtyard. A: CromaLux light, B: Metal halide lamp (positive control). 1: location of the insect trap beside the CromaLux lamp, 2: location of the insect trap beside the metal halide lamp, 3: location of the insect trap in the unilluminated area. *: sheets used to prevent interference between both light systems. Aerial photographs from PNOA 2020 © CNIG.

**Figure 3:**
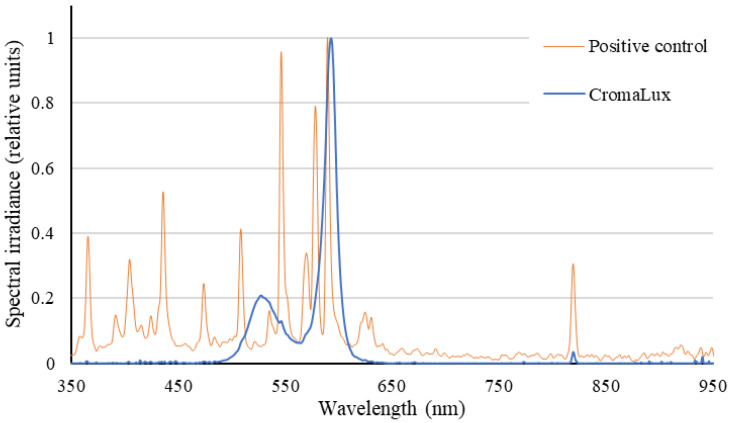
Normalised spectra of the CromaLux lamp with a CCT of 3000 K (blue line) and the metal halide lamp with a CCT of 4668 K (orange line). No significant differences were found between the mean values of light intensity in luxes of both lamps in the surrounding areas where the insects were trapped. The spectral sensitivity range of insects is 330–640 nm [38].

**Figure 4:**
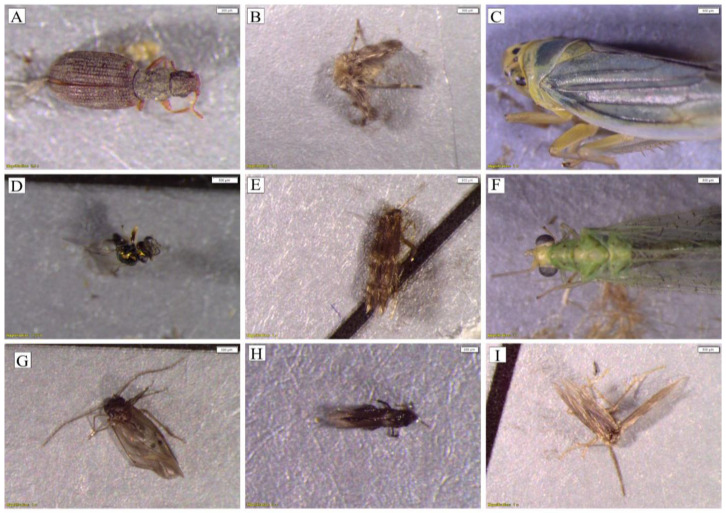
Photographs of some of the insects captured. (**A**) Coleoptera (Latridiidae), (**B**) Diptera (*Psychodidae*), (**C**) Hemiptera (*Cicadella viridis*), (**D**) Hymenoptera (Chrysididae), (**E**) Lepidoptera, (**F**) Neuroptera (*Chrysoperla carnea*), (**G**) Psocoptera (Psocomorpha), (**H**) Thysanoptera, and (**I**) *Trichoptera* (Hydroptilidae).

**Figure 5:**
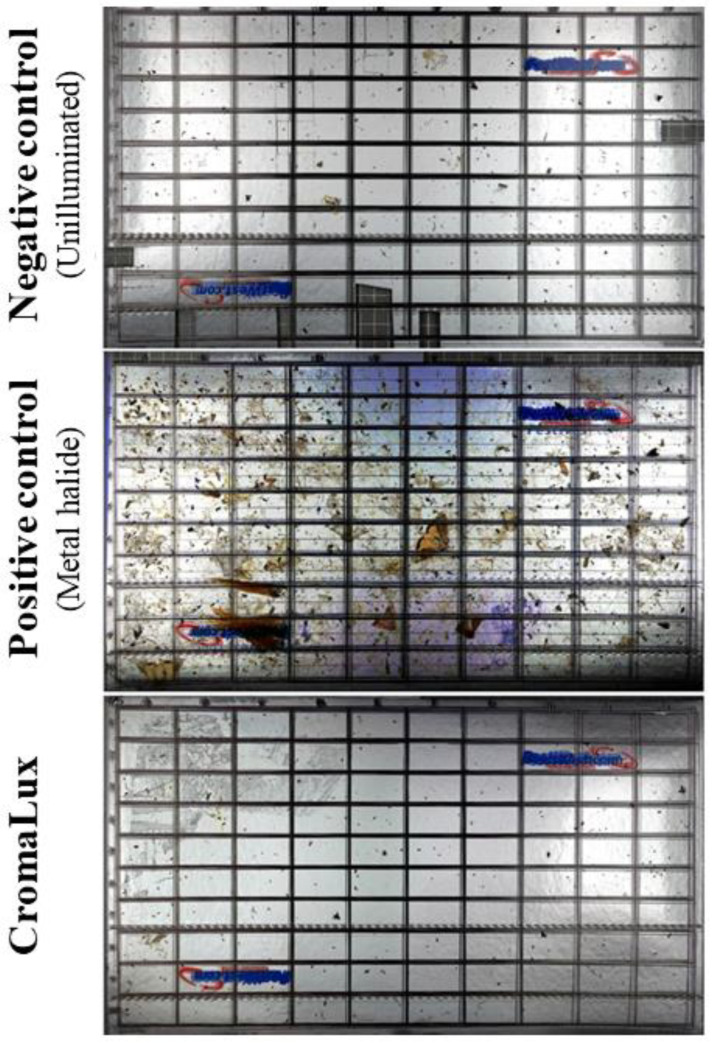
Overlapped photographs of all sticky board traps for the three lighting systems.

**Figure 6:**
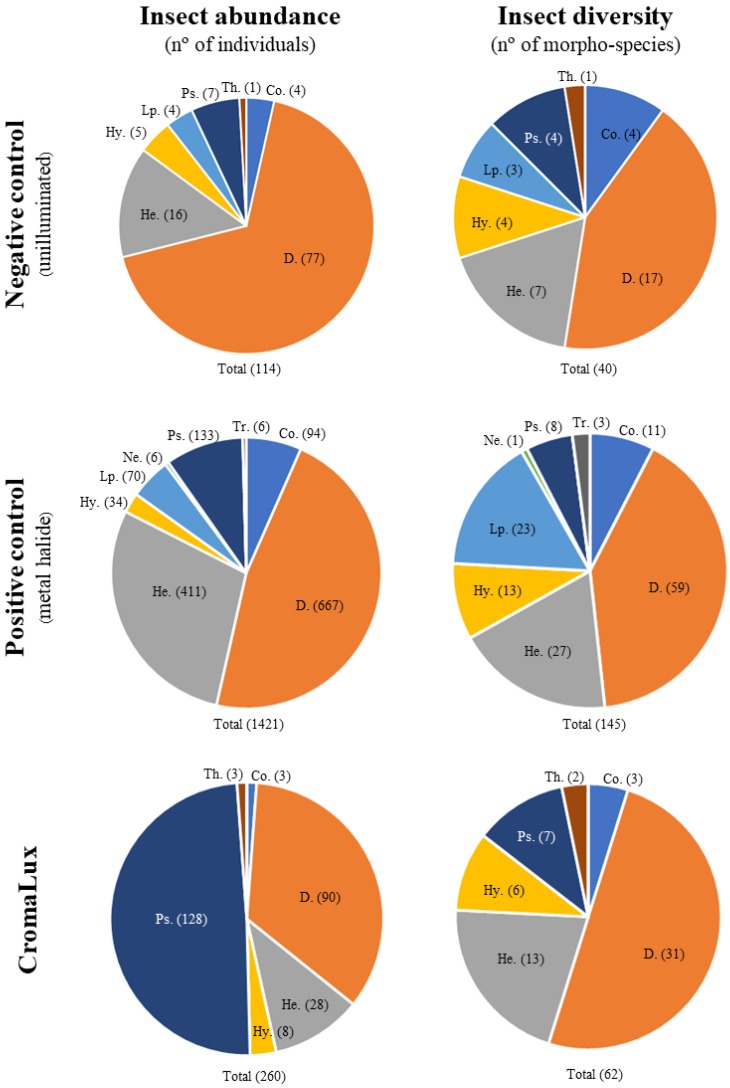
Insect abundance and insect biodiversity captured beside the three lighting systems. Co: Coleoptera, D.: Diptera, He.: Hemiptera, Hy.: Hymenoptera, Lp.: Lepidoptera, Ne.: Neuroptera, Ps.: Psocoptera, Th.: Thysanoptera, Tr.: *Trichoptera*.

**Figure 7:**
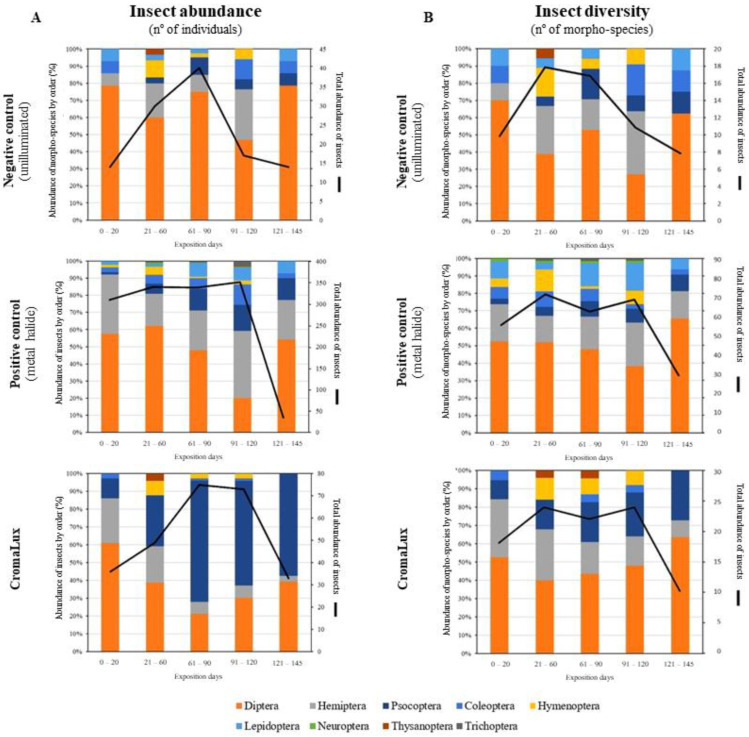
Temporal succession of insect abundance (**A**) and insect diversity (**B**) corresponding to the three lighting conditions during the five study periods (see Table 1). The black line represents the total abundance of insects (**A**) and of insect morphospecies (**B**). The numbers in (**B**) indicate the Shannon–Wiener index for each time period.

In this study, we tested how CromaLux (Figure 1, designed for illuminating monuments because its biostatic activity against phototrophic organisms) a warm LED light of 3000 K and absence of UV-blue light (Figure 3) impacts nocturnal biodiversity (considering the insect community as the reference group). For comparative purposes, we also tested a metal halide lamp of the type currently in use to illuminate monuments (4668 K, Figure 2) and no artificial lighting. There were no significant differences in abundance or composition of the insects captured in the traps beside the CromaLux LED light and the in the unilluminated area, but the impact was significantly lower than that caused by the metal halide lamps (Figure 5, Figure 6 and Figure 7). Although the heat emitted by metal halide lamp may be expected to contribute to attracting insects, the temperatures of the sticky board traps beside the metal halide and CromaLux lamps were almost identical (12.25 ± 2.42 °C vs. 12.13 ± 2.53 °C, respectively). Differences in CCT is another possible factor; however, previous studies have shown that colour temperature and insect attraction are not necessarily related, because the spectral composition also influences phototaxis for nocturnal insects [45]. Thus, CCT per se is not the reason for the attraction, and it is the differences in lighting spectra that cause the difference as confirmed by the statistical analysis.

It is surprising the small number of captured Lepidoptera (4% of the total), particularly when considering that baiting the light is the best way to catch many kinds of butterflies and moths. In our opinion, the three main reasons for this to happen are: (i) the location of the experiment in an inner courtyard inside an urban area (instead of a wooded area), (ii) the decline of Lepidoptera in recent decades, especially in urban environments and caused in large part by the anthropogenic light, which has been shown to lead, for example, to negative population trends of some European moths [17], and (iii) the insufficient strength of the sticky board traps used to trap the insects for those larger and heavier. Indeed, we have observed throughout the experiment in the traps signs of impact (remains of scales, tarsal segments, etc.) associated with larger and heavier insects, such as moths of a certain size and larger genera of Coleoptera, which have managed to escape after being partially adhered.

Regarding the insects captured and their relationship with the three lighting conditions, nine insect orders expected to be found in an urban environment [46] were identified, although they were less abundant in June and October (Figure 7) when mean precipitation was higher and average temperature was lower (Table 1). The insect capture followed a trend that can be explained in terms of the spectral sensitivity of the visual systems.

There is some variation in dipteran spectral sensitivity, ranging from UV and green photoreceptors (e.g., *Aedes*) to UV, blue, blue–green, and green photoreceptors (e.g., *Musca*) [47]. In the present study, members of the order Diptera (the most abundant order here) were particularly attracted to the light emitted by the metal halide lamp. This concurs with the findings of previous studies. For example, *Tanytarsus barbitarses* (Diptera: Chironomidae) was more strongly attracted to white light than to green light, with peak attraction near the ultraviolet region at 370–400 nm [48] and *Lutzomyia longipalpis* (Diptera: Psychodidae) was mainly attracted to the UV wavelengths (<400 nm) rather than to the blue–green wavelengths [49].

Hemiptera typically responds to the ultraviolet (330–350 nm), blue (405–490 nm), and green (520–560 nm) wavelengths [38], emitted in the present study by the metal halide lamp to which they were most attracted, but some species also exhibit a unimodal response centred in the green wavelengths (emitted by both lamps), such as the aphid *Acyrthosiphon pisum* [50] and the bedbug *Cimex lectularius* [51].

Psocids (Psocoptera) have shown spectral sensitivity to UV and green wavelengths. *Liposcelis paeta* (Psocoptera: Liposcelididae) females responded positively to two UV wavelengths (351 and 400 nm) and to green light (527 nm) [52], and 351 nm UV light triggers a very strong phototaxis response from *Liposcelis bostrychophila* (Psocoptera: Liposcelididae) adults [53]. Sensitivity to those parts of the spectrum is common in most arthropods [37]. In the present study, the number captured of members of the order Psocoptera was similar beside the CromaLux and metal halide lamps (Figure 6). This could suggest that there was no great impact on the presence or absence of UV-blue wavelengths (or other spectral/intensity differences between the lamps) on phototaxis in the specimens of this group trapped here. In this regard, although the peaks are different, the total photon count may be similar in the green-wavelength portion of both lamps (Figure 3), and this could generate the same attraction in the Psocoptera group of the present study. In addition, the similar responses could also be explained by a lack of input from any UV or blue photoreceptors (if present) to the phototaxis system of the Psocoptera members trapped.

Some members of Carabidae family (Coleoptera) have two peaks (UV and green) and others have been shown to have four peaks of spectral sensitivity (UV, blue, green, and red) [38]. The pollen beetle *Meligethes aeneus* (Coleoptera: Nitidulidae) displays a peak in spectral sensitivity at around 550 nm (green region) and a secondary sensitivity peak is observed in the UV range (370 nm), but not in the blue region [50]. In the present study, Coleoptera individuals were particularly attracted to the light emitted by the metal halide lamp, suggesting an influence, at least of the UV light difference between both lamps, on these individuals.

Lepidoptera are strongly attracted to the UV and blue part of the spectrum [54]. Some noctuid moths, such as *N. pronuba*, appear to have additional sensitivity to red light [40] and thus may be affected by traditional warm LED illumination (~3000 K), which often emits in the red part of the spectrum. The reproductive cycle of nocturnal Lepidoptera (or moths) is particularly sensitive to light pollution [17], which can have important consequences for the conservation of populations. The lack of attraction of the CromaLux to Lepidoptera specimens due to the spectral composition of the light emitted makes this an ideal system to prevent the negative effects of monumental lighting on these populations.

The Shannon–Wiener index values showed that the CromaLux lamps did not increase the diversity of morphospecies attracted to the light, resulting in a lower effect on the nocturnal biodiversity of this type of lamp relative to metal halide lamps. The findings are consistent with previous studies with streetlights, such as [14,55], who reported the transition to LED from traditional light technologies reduces the impact on aerial nocturnal insects, and also with those of [18,45], who reported that specific selection of the spectra of the LED changes the overall attraction to the light. The present study went a step further by specifically selecting an adequate spectrum for monumental illumination.

## 5. Conclusions

In summary, the study findings show that an LED lamp emitting a combination of amber and green light (as shown in Figure 3) effectively reduced both the abundance and diversity of insects attracted to the light source compared to traditional light sources such as metal halide lamps. This effect has been demonstrated for almost all insect orders trapped, especially in Diptera, Lepidoptera, Coleoptera, Hemiptera, and Hymenoptera. On the contrary, Psocoptera showed a similar attraction to the CromaLux and metal halide lamps, a phenomenon whose causes deserve further investigation. As expected, Diptera were the most diverse and abundant insects in all samples, but the abundance of Lepidoptera was unexpectedly low (4%), which, regardless of certain methodological limitations or site-dependent effects, is in line with the worldwide evidence of the progressive decline of populations of this group.

Conscious selection of the light spectra used for ornamental illumination will reduce the impact on insect biodiversity, relative to more traditional lighting systems, while still showcasing the artistic and aesthetic features of heritage buildings (Figure 1). Illumination of monuments is also part of night-time street lighting and thus contributes to public safety. The study provides reference findings regarding the application of principles of good practices leading to possible regulatory and legal solutions and the incorporation of specific measures for artificial lighting of monuments or urban structures.

## Figures and Tables

**Table 1:** Sampling (clustered in five similar time periods) carried out between 1 June and 20 October 2021 and corresponding meteorological data, extracted from METEOGALICIA. Available online: www.meteogalicia.gal, accessed on 13 December 2022.

Period	N° of Replacements	Rain (L m^−2^)	Sunshine Duration (h)	Mean Relative Humidity (%)	Average Air Temperature (°C)
[1] 1–21 June	3	3.18 ± 6.98	8.10 ± 4.93	78.95 ± 7.91	16.25 ± 3.32
[2] 21–30 June	3	0.58 ± 1.38	7.16 ± 4.38	80.56 ± 7.62	16.93 ± 2.23
[3] 30–28 July	4	0.44 ± 1.30	8.15 ± 3.41	81.25 ± 5.13	18.30 ± 1.54
[4] 28–26 August	3	2.76 ± 5.01	6.10 ± 3.33	83.03 ± 6.96	17.32 ± 2.08
[5] 26 September–20 October	2	4.27 ± 8.58	5.12 ± 3.69	87.88 ± 6.31	14.72 ± 1.26

**Table 2:** Number of specimens and morphospecies of the orders trapped throughout the experiment.

Order	% of Insects Trapped	N° Specimens	N° of Morphospecies
Diptera	47.1	842	65
Hemiptera	25.0	457	28
Psocoptera	15.0	268	8
Coleoptera	5.5	100	14
Lepidoptera	4.0	74	24
Hymenoptera	2.6	47	14
Neuroptera	0.3	6	1
*Trichoptera*	0.3	6	3
Thysanoptera	0.2	4	3
Total	100	1804	160

## Data Availability

During the present research entities, the datasets and results gathered and generated from the analysis after the identification of the specimens recollected are available from the corresponding author upon reasonable request.

## Supplementary Materials

The following supporting information can be downloaded at: https://www.mdpi.com/article/10.3390/insects13121153/s1, Table S1: Taxonomic identification of the insects trapped throughout the experiment; Table S2: Data used for the calculation of the Shannon-Wiener index.
