# Supplementary material for: Attraction of Insects to Ornamental Lighting Used on Cultural Heritage Buildings: A Case Study in an Urban Area
Source: Insects. 2022 Dec 14;13(12):1153. doi: 10.3390/insects13121153 (PMC9783376; doi:10.3390/insects13121153)
Supplement: Supplementary file 1 [file insects-13-01153-s001.zip › insects-2053305-supplementary.pdf]

**Table S1.** Taxonomic identification of the insects trapped throughout the experiment.

| Order | Suborder | Family | Genera/species | Number of morpho-species |
|---|---|---|---|---|
| Coleoptera (14) | Adephaga (2) | Carabidae | | 2 |
| | Polyphaga (12) | Bostrichidae | *Schistocerus* | 1 |
| | | Chrysomelidae | | 1 |
| | | Cryptophagidae | | 1 |
| | | Latridiidae | | 2 |
| | | Nitidulidae | *Cybocephalus* | 1 |
| | | | | 1 |
| | | Ptiliidae | | 1 |
| | | Silvanidae | | 1 |
| | | Staphylinidae | | 2 |
| | | Tenebrionidae | | 1 |
| Diptera (65) | Nematocera (22) | Chironomidae | | 3 |
| | | Psychodidae | | 4 |
| | | Tipuloidea (Superfamily) | | 5 |
| | | | | 10 |
| | Schizophora (7) | Muscidae | | 6 |
| | | | | 1 |

| | | | | |
|---|---|---|---|---|
| | | | | 36 |
| Hemiptera (28) | Cicadomorpha (18) | Cicadellidae | *Cicadella viridis* | 1 |
| | | | | 17 |
| | Heteroptera (5) | Miridae | | 5 |
| | Sternorrhyncha (5) | Aphididae | | 5 |
| Hymenoptera (14) | Apocrita (4) | Chrysididae | | 1 |
| | | Formicidae | | 2 |
| | | Ichneumonidae | | 1 |
| | Symphyta (4) | Cephidae | | 2 |
| | | | | 2 |
| | | | | 6 |
| Lepidoptera (24) | Glossata (2) | Noctuidae | *Lacanobia oleracea* | 1 |
| | | | *Noctua pronuba* | 1 |
| | | | | 21 |
| Neuroptera (1) | Hemerobiiformia (1) | Chrysopidae | *Chrysoperla carnea* | 1 |
| Psocoptera (8) | Psocomorpha (8) | | | 8 |
| Thysanoptera (3) | | | | 3 |
| Trichoptera (3) | Integripalpia (1) | Lepidostomatidae | *Lepidostoma basale* | 1 |
| | Spicipalpia (1) | Hidroptilidae | | 1 |

| | | |
|---|---|---|
| Philopotamidae | *Wormaldia* | 1 |

**Table S2.** Data used for the calculation of the Shannon-Wiener index.

| Order | Morpho-species | Negative control (Unilluminated) | | | | Positive control (Metal halide) | | | | CromaLux | | | |
|---|---|---|---|---|---|---|---|---|---|---|---|---|---|
| | | Nº of individuals | Pi | lnPi | Pi*lnPi | Nº of individuals | Pi | lnPi | Pi*lnPi | Nº of individuals | Pi | lnPi | Pi*lnPi |
| Coleoptera | Coleoptera 1 | 1 | 8.77E-03 | -4.74E+00 | -4.15E-02 | 13 | 9.15E-03 | -4.69E+00 | -4.30E-02 | 0 | 0.00E+00 | 0.00E+00 | 0.00E+00 |
| | Coleoptera 2 | 0 | 0.00E+00 | 0.00E+00 | 0.00E+00 | 0 | 0.00E+00 | 0.00E+00 | 0.00E+00 | 1 | 3.85E-03 | -5.56E+00 | -2.14E-02 |
| | Coleoptera 3 | 1 | 8.77E-03 | -4.74E+00 | -4.15E-02 | 62 | 4.37E-02 | -3.13E+00 | -1.37E-01 | 1 | 3.85E-03 | -5.56E+00 | -2.14E-02 |
| | Coleoptera 4 | 0 | 0.00E+00 | 0.00E+00 | 0.00E+00 | 2 | 1.41E-03 | -6.57E+00 | -9.25E-03 | 0 | 0.00E+00 | 0.00E+00 | 0.00E+00 |
| | Coleoptera 5 | 0 | 0.00E+00 | 0.00E+00 | 0.00E+00 | 2 | 1.41E-03 | -6.57E+00 | -9.25E-03 | 0 | 0.00E+00 | 0.00E+00 | 0.00E+00 |
| | Coleoptera 6 | 0 | 0.00E+00 | 0.00E+00 | 0.00E+00 | 3 | 2.11E-03 | -6.16E+00 | -1.30E-02 | 0 | 0.00E+00 | 0.00E+00 | 0.00E+00 |
| | Coleoptera 7 | 1 | 8.77E-03 | -4.74E+00 | -4.15E-02 | 5 | 3.52E-03 | -5.65E+00 | -1.99E-02 | 0 | 0.00E+00 | 0.00E+00 | 0.00E+00 |
| | Coleoptera 8 | 0 | 0.00E+00 | 0.00E+00 | 0.00E+00 | 2 | 1.41E-03 | -6.57E+00 | -9.25E-03 | 0 | 0.00E+00 | 0.00E+00 | 0.00E+00 |
| | Coleoptera 9 | 0 | 0.00E+00 | 0.00E+00 | 0.00E+00 | 1 | 7.04E-04 | -7.26E+00 | -5.11E-03 | 0 | 0.00E+00 | 0.00E+00 | 0.00E+00 |
| | Coleoptera 10 | 0 | 0.00E+00 | 0.00E+00 | 0.00E+00 | 2 | 1.41E-03 | -6.57E+00 | -9.25E-03 | 0 | 0.00E+00 | 0.00E+00 | 0.00E+00 |
| | Coleoptera 11 | 0 | 0.00E+00 | 0.00E+00 | 0.00E+00 | 0 | 0.00E+00 | 0.00E+00 | 0.00E+00 | 1 | 3.85E-03 | -5.56E+00 | -2.14E-02 |
| | Coleoptera 12 | 0 | 0.00E+00 | 0.00E+00 | 0.00E+00 | 1 | 7.04E-04 | -7.26E+00 | -5.11E-03 | 0 | 0.00E+00 | 0.00E+00 | 0.00E+00 |
| | Coleoptera 13 | 0 | 0.00E+00 | 0.00E+00 | 0.00E+00 | 1 | 7.04E-04 | -7.26E+00 | -5.11E-03 | 0 | 0.00E+00 | 0.00E+00 | 0.00E+00 |
| | Coleoptera 14 | 1 | 8.77E-03 | -4.74E+00 | -4.15E-02 | 0 | 0.00E+00 | 0.00E+00 | 0.00E+00 | 0 | 0.00E+00 | 0.00E+00 | 0.00E+00 |
| Diptera | Diptera 1 | 1 | 8.77E-03 | -4.74E+00 | -4.15E-02 | 2 | 1.41E-03 | -6.57E+00 | -9.25E-03 | 2 | 7.69E-03 | -4.87E+00 | -3.74E-02 |
| | Diptera 2 | 8 | 7.02E-02 | -2.66E+00 | -1.86E-01 | 28 | 1.97E-02 | -3.93E+00 | -7.74E-02 | 1 | 3.85E-03 | -5.56E+00 | -2.14E-02 |
| | Diptera 3 | 42 | 3.68E-01 | -9.99E-01 | -3.68E-01 | 178 | 1.25E-01 | -2.08E+00 | -2.60E-01 | 28 | 1.08E-01 | -2.23E+00 | -2.40E-01 |
| | Diptera 4 | 1 | 8.77E-03 | -4.74E+00 | -4.15E-02 | 4 | 2.82E-03 | -5.87E+00 | -1.65E-02 | 0 | 0.00E+00 | 0.00E+00 | 0.00E+00 |
| | Diptera 5 | 1 | 8.77E-03 | -4.74E+00 | -4.15E-02 | 20 | 1.41E-02 | -4.26E+00 | -6.00E-02 | 3 | 1.15E-02 | -4.46E+00 | -5.15E-02 |
| | Diptera 6 | 0 | 0.00E+00 | 0.00E+00 | 0.00E+00 | 21 | 1.48E-02 | -4.21E+00 | -6.23E-02 | 1 | 3.85E-03 | -5.56E+00 | -2.14E-02 |
| | Diptera 7 | 0 | 0.00E+00 | 0.00E+00 | 0.00E+00 | 4 | 2.82E-03 | -5.87E+00 | -1.65E-02 | 0 | 0.00E+00 | 0.00E+00 | 0.00E+00 |
| | Diptera 8 | 5 | 4.39E-02 | -3.13E+00 | -1.37E-01 | 25 | 1.76E-02 | -4.04E+00 | -7.11E-02 | 11 | 4.23E-02 | -3.16E+00 | -1.34E-01 |
| | Diptera 9 | 1 | 8.77E-03 | -4.74E+00 | -4.15E-02 | 3 | 2.11E-03 | -6.16E+00 | -1.30E-02 | 0 | 0.00E+00 | 0.00E+00 | 0.00E+00 |
| | Diptera 10 | 0 | 0.00E+00 | 0.00E+00 | 0.00E+00 | 5 | 3.52E-03 | -5.65E+00 | -1.99E-02 | 0 | 0.00E+00 | 0.00E+00 | 0.00E+00 |
| | Diptera 11 | 0 | 0.00E+00 | 0.00E+00 | 0.00E+00 | 40 | 2.82E-02 | -3.57E+00 | -1.01E-01 | 2 | 7.69E-03 | -4.87E+00 | -3.74E-02 |

**Table S2.** Data used for the calculation of the Shannon-Wiener index.

| | | | | | | | | | | | | |
|---|---|---|---|---|---|---|---|---|---|---|---|---|
| Diptera 12 | 4 | 3.51E-02 | -3.35E+00 | -1.18E-01 | 12 | 8.45E-03 | -4.77E+00 | -4.03E-02 | 2 | 7.69E-03 | -4.87E+00 | -3.74E-02 |
| Diptera 13 | 0 | 0.00E+00 | 0.00E+00 | 0.00E+00 | 19 | 1.34E-02 | -4.31E+00 | -5.77E-02 | 1 | 3.85E-03 | -5.56E+00 | -2.14E-02 |
| Diptera 14 | 0 | 0.00E+00 | 0.00E+00 | 0.00E+00 | 8 | 5.63E-03 | -5.18E+00 | -2.92E-02 | 1 | 3.85E-03 | -5.56E+00 | -2.14E-02 |
| Diptera 15 | 0 | 0.00E+00 | 0.00E+00 | 0.00E+00 | 3 | 2.11E-03 | -6.16E+00 | -1.30E-02 | 1 | 3.85E-03 | -5.56E+00 | -2.14E-02 |
| Diptera 16 | 0 | 0.00E+00 | 0.00E+00 | 0.00E+00 | 4 | 2.82E-03 | -5.87E+00 | -1.65E-02 | 0 | 0.00E+00 | 0.00E+00 | 0.00E+00 |
| Diptera 17 | 1 | 8.77E-03 | -4.74E+00 | -4.15E-02 | 8 | 5.63E-03 | -5.18E+00 | -2.92E-02 | 0 | 0.00E+00 | 0.00E+00 | 0.00E+00 |
| Diptera 18 | 1 | 8.77E-03 | -4.74E+00 | -4.15E-02 | 13 | 9.15E-03 | -4.69E+00 | -4.30E-02 | 0 | 0.00E+00 | 0.00E+00 | 0.00E+00 |
| Diptera 19 | 0 | 0.00E+00 | 0.00E+00 | 0.00E+00 | 26 | 1.83E-02 | -4.00E+00 | -7.32E-02 | 3 | 1.15E-02 | -4.46E+00 | -5.15E-02 |
| Diptera 20 | 0 | 0.00E+00 | 0.00E+00 | 0.00E+00 | 12 | 8.45E-03 | -4.77E+00 | -4.03E-02 | 1 | 3.85E-03 | -5.56E+00 | -2.14E-02 |
| Diptera 21 | 0 | 0.00E+00 | 0.00E+00 | 0.00E+00 | 11 | 7.75E-03 | -4.86E+00 | -3.77E-02 | 1 | 3.85E-03 | -5.56E+00 | -2.14E-02 |
| Diptera 22 | 0 | 0.00E+00 | 0.00E+00 | 0.00E+00 | 3 | 2.11E-03 | -6.16E+00 | -1.30E-02 | 0 | 0.00E+00 | 0.00E+00 | 0.00E+00 |
| Dípetra 23 | 5 | 4.39E-02 | -3.13E+00 | -1.37E-01 | 5 | 3.52E-03 | -5.65E+00 | -1.99E-02 | 0 | 0.00E+00 | 0.00E+00 | 0.00E+00 |
| Diptera 24 | 0 | 0.00E+00 | 0.00E+00 | 0.00E+00 | 8 | 5.63E-03 | -5.18E+00 | -2.92E-02 | 0 | 0.00E+00 | 0.00E+00 | 0.00E+00 |
| Diptera 25 | 0 | 0.00E+00 | 0.00E+00 | 0.00E+00 | 2 | 1.41E-03 | -6.57E+00 | -9.25E-03 | 0 | 0.00E+00 | 0.00E+00 | 0.00E+00 |
| Diptera 26 | 0 | 0.00E+00 | 0.00E+00 | 0.00E+00 | 15 | 1.06E-02 | -4.55E+00 | -4.81E-02 | 3 | 1.15E-02 | -4.46E+00 | -5.15E-02 |
| Diptera 27 | 0 | 0.00E+00 | 0.00E+00 | 0.00E+00 | 0 | 0.00E+00 | 0.00E+00 | 0.00E+00 | 1 | 3.85E-03 | -5.56E+00 | -2.14E-02 |
| Diptera 28 | 0 | 0.00E+00 | 0.00E+00 | 0.00E+00 | 7 | 4.93E-03 | -5.31E+00 | -2.62E-02 | 0 | 0.00E+00 | 0.00E+00 | 0.00E+00 |
| Diptera 29 | 0 | 0.00E+00 | 0.00E+00 | 0.00E+00 | 1 | 7.04E-04 | -7.26E+00 | -5.11E-03 | 0 | 0.00E+00 | 0.00E+00 | 0.00E+00 |
| Diptera 30 | 0 | 0.00E+00 | 0.00E+00 | 0.00E+00 | 19 | 1.34E-02 | -4.31E+00 | -5.77E-02 | 0 | 0.00E+00 | 0.00E+00 | 0.00E+00 |
| Diptera 31 | 1 | 8.77E-03 | -4.74E+00 | -4.15E-02 | 17 | 1.20E-02 | -4.43E+00 | -5.30E-02 | 0 | 0.00E+00 | 0.00E+00 | 0.00E+00 |
| Diptera 32 | 1 | 8.77E-03 | -4.74E+00 | -4.15E-02 | 7 | 4.93E-03 | -5.31E+00 | -2.62E-02 | 1 | 3.85E-03 | -5.56E+00 | -2.14E-02 |
| Diptera 33 | 0 | 0.00E+00 | 0.00E+00 | 0.00E+00 | 6 | 4.23E-03 | -5.47E+00 | -2.31E-02 | 1 | 3.85E-03 | -5.56E+00 | -2.14E-02 |
| Diptera 34 | 0 | 0.00E+00 | 0.00E+00 | 0.00E+00 | 3 | 2.11E-03 | -6.16E+00 | -1.30E-02 | 0 | 0.00E+00 | 0.00E+00 | 0.00E+00 |
| Diptera 35 | 0 | 0.00E+00 | 0.00E+00 | 0.00E+00 | 4 | 2.82E-03 | -5.87E+00 | -1.65E-02 | 2 | 7.69E-03 | -4.87E+00 | -3.74E-02 |
| Diptera 36 | 0 | 0.00E+00 | 0.00E+00 | 0.00E+00 | 3 | 2.11E-03 | -6.16E+00 | -1.30E-02 | 1 | 3.85E-03 | -5.56E+00 | -2.14E-02 |
| Diptera 37 | 0 | 0.00E+00 | 0.00E+00 | 0.00E+00 | 1 | 7.04E-04 | -7.26E+00 | -5.11E-03 | 3 | 1.15E-02 | -4.46E+00 | -5.15E-02 |
| Diptera 38 | 0 | 0.00E+00 | 0.00E+00 | 0.00E+00 | 5 | 3.52E-03 | -5.65E+00 | -1.99E-02 | 1 | 3.85E-03 | -5.56E+00 | -2.14E-02 |
| Diptera 39 | 0 | 0.00E+00 | 0.00E+00 | 0.00E+00 | 5 | 3.52E-03 | -5.65E+00 | -1.99E-02 | 0 | 0.00E+00 | 0.00E+00 | 0.00E+00 |

**Table S2.** Data used for the calculation of the Shannon-Wiener index.

| | | | | | | | | | | | | | |
|---|---|---|---|---|---|---|---|---|---|---|---|---|---|
| | Diptera 40 | 0 | 0.00E+00 | 0.00E+00 | 0.00E+00 | 2 | 1.41E-03 | -6.57E+00 | -9.25E-03 | 0 | 0.00E+00 | 0.00E+00 | 0.00E+00 |
| | Diptera 41 | 1 | 8.77E-03 | -4.74E+00 | -4.15E-02 | 13 | 9.15E-03 | -4.69E+00 | -4.30E-02 | 0 | 0.00E+00 | 0.00E+00 | 0.00E+00 |
| | Diptera 42 | 0 | 0.00E+00 | 0.00E+00 | 0.00E+00 | 7 | 4.93E-03 | -5.31E+00 | -2.62E-02 | 0 | 0.00E+00 | 0.00E+00 | 0.00E+00 |
| | Diptera 43 | 0 | 0.00E+00 | 0.00E+00 | 0.00E+00 | 2 | 1.41E-03 | -6.57E+00 | -9.25E-03 | 0 | 0.00E+00 | 0.00E+00 | 0.00E+00 |
| | Diptera 44 | 0 | 0.00E+00 | 0.00E+00 | 0.00E+00 | 7 | 4.93E-03 | -5.31E+00 | -2.62E-02 | 0 | 0.00E+00 | 0.00E+00 | 0.00E+00 |
| | Diptera 45 | 0 | 0.00E+00 | 0.00E+00 | 0.00E+00 | 4 | 2.82E-03 | -5.87E+00 | -1.65E-02 | 0 | 0.00E+00 | 0.00E+00 | 0.00E+00 |
| | Diptera 46 | 0 | 0.00E+00 | 0.00E+00 | 0.00E+00 | 1 | 7.04E-04 | -7.26E+00 | -5.11E-03 | 0 | 0.00E+00 | 0.00E+00 | 0.00E+00 |
| | Diptera 47 | 2 | 1.75E-02 | -4.04E+00 | -7.09E-02 | 8 | 5.63E-03 | -5.18E+00 | -2.92E-02 | 5 | 1.92E-02 | -3.95E+00 | -7.60E-02 |
| | Diptera 48 | 0 | 0.00E+00 | 0.00E+00 | 0.00E+00 | 1 | 7.04E-04 | -7.26E+00 | -5.11E-03 | 0 | 0.00E+00 | 0.00E+00 | 0.00E+00 |
| | Diptera 49 | 0 | 0.00E+00 | 0.00E+00 | 0.00E+00 | 3 | 2.11E-03 | -6.16E+00 | -1.30E-02 | 0 | 0.00E+00 | 0.00E+00 | 0.00E+00 |
| | Diptera 50 | 0 | 0.00E+00 | 0.00E+00 | 0.00E+00 | 5 | 3.52E-03 | -5.65E+00 | -1.99E-02 | 1 | 3.85E-03 | -5.56E+00 | -2.14E-02 |
| | Diptera 51 | 1 | 8.77E-03 | -4.74E+00 | -4.15E-02 | 0 | 0.00E+00 | 0.00E+00 | 0.00E+00 | 1 | 3.85E-03 | -5.56E+00 | -2.14E-02 |
| | Diptera 52 | 0 | 0.00E+00 | 0.00E+00 | 0.00E+00 | 6 | 4.23E-03 | -5.47E+00 | -2.31E-02 | 0 | 0.00E+00 | 0.00E+00 | 0.00E+00 |
| | Diptera 53 | 0 | 0.00E+00 | 0.00E+00 | 0.00E+00 | 21 | 1.48E-02 | -4.21E+00 | -6.23E-02 | 3 | 1.15E-02 | -4.46E+00 | -5.15E-02 |
| | Diptera 54 | 0 | 0.00E+00 | 0.00E+00 | 0.00E+00 | 8 | 5.63E-03 | -5.18E+00 | -2.92E-02 | 1 | 3.85E-03 | -5.56E+00 | -2.14E-02 |
| | Diptera 55 | 1 | 8.77E-03 | -4.74E+00 | -4.15E-02 | 7 | 4.93E-03 | -5.31E+00 | -2.62E-02 | 4 | 1.54E-02 | -4.17E+00 | -6.42E-02 |
| | Diptera 56 | 0 | 0.00E+00 | 0.00E+00 | 0.00E+00 | 2 | 1.41E-03 | -6.57E+00 | -9.25E-03 | 0 | 0.00E+00 | 0.00E+00 | 0.00E+00 |
| | Diptera 57 | 0 | 0.00E+00 | 0.00E+00 | 0.00E+00 | 5 | 3.52E-03 | -5.65E+00 | -1.99E-02 | 1 | 3.85E-03 | -5.56E+00 | -2.14E-02 |
| | Diptera 58 | 0 | 0.00E+00 | 0.00E+00 | 0.00E+00 | 2 | 1.41E-03 | -6.57E+00 | -9.25E-03 | 1 | 3.85E-03 | -5.56E+00 | -2.14E-02 |
| | Diptera 59 | 0 | 0.00E+00 | 0.00E+00 | 0.00E+00 | 2 | 1.41E-03 | -6.57E+00 | -9.25E-03 | 0 | 0.00E+00 | 0.00E+00 | 0.00E+00 |
| | Diptera 60 | 0 | 0.00E+00 | 0.00E+00 | 0.00E+00 | 3 | 2.11E-03 | -6.16E+00 | -1.30E-02 | 0 | 0.00E+00 | 0.00E+00 | 0.00E+00 |
| | Diptera 61 | 0 | 0.00E+00 | 0.00E+00 | 0.00E+00 | 1 | 7.04E-04 | -7.26E+00 | -5.11E-03 | 2 | 7.69E-03 | -4.87E+00 | -3.74E-02 |
| Hemiptera | Hemiptera 1 | 2 | 1.75E-02 | -4.04E+00 | -7.09E-02 | 86 | 6.06E-02 | -2.80E+00 | -1.70E-01 | 8 | 3.08E-02 | -3.48E+00 | -1.07E-01 |
| | Hemiptera 2 | 0 | 0.00E+00 | 0.00E+00 | 0.00E+00 | 3 | 2.11E-03 | -6.16E+00 | -1.30E-02 | 1 | 3.85E-03 | -5.56E+00 | -2.14E-02 |
| | Hemiptera 3 | 0 | 0.00E+00 | 0.00E+00 | 0.00E+00 | 50 | 3.52E-02 | -3.35E+00 | -1.18E-01 | 5 | 1.92E-02 | -3.95E+00 | -7.60E-02 |
| | Hemiptera 4 | 6 | 5.26E-02 | -2.94E+00 | -1.55E-01 | 29 | 2.04E-02 | -3.89E+00 | -7.95E-02 | 2 | 7.69E-03 | -4.87E+00 | -3.74E-02 |
| | Hemiptera 5 | 3 | 2.63E-02 | -3.64E+00 | -9.57E-02 | 3 | 2.11E-03 | -6.16E+00 | -1.30E-02 | 3 | 1.15E-02 | -4.46E+00 | -5.15E-02 |
| | Hemiptera 6 | 0 | 0.00E+00 | 0.00E+00 | 0.00E+00 | 21 | 1.48E-02 | -4.21E+00 | -6.23E-02 | 0 | 0.00E+00 | 0.00E+00 | 0.00E+00 |

**Table S2.** Data used for the calculation of the Shannon-Wiener index.

| | | | | | | | | | | | | | |
|---|---|---|---|---|---|---|---|---|---|---|---|---|---|
| | Hemiptera 7 | 2 | 1.75E-02 | -4.04E+00 | -7.09E-02 | 41 | 2.89E-02 | -3.54E+00 | -1.02E-01 | 2 | 7.69E-03 | -4.87E+00 | -3.74E-02 |
| | Hemiptera 8 | 0 | 0.00E+00 | 0.00E+00 | 0.00E+00 | 4 | 2.82E-03 | -5.87E+00 | -1.65E-02 | 1 | 3.85E-03 | -5.56E+00 | -2.14E-02 |
| | Hemiptera 9 | 0 | 0.00E+00 | 0.00E+00 | 0.00E+00 | 61 | 4.30E-02 | -3.15E+00 | -1.35E-01 | 1 | 3.85E-03 | -5.56E+00 | -2.14E-02 |
| | Hemiptera 10 | 0 | 0.00E+00 | 0.00E+00 | 0.00E+00 | 5 | 3.52E-03 | -5.65E+00 | -1.99E-02 | 0 | 0.00E+00 | 0.00E+00 | 0.00E+00 |
| | Hemiptera 11 | 1 | 8.77E-03 | -4.74E+00 | -4.15E-02 | 3 | 2.11E-03 | -6.16E+00 | -1.30E-02 | 1 | 3.85E-03 | -5.56E+00 | -2.14E-02 |
| | Hemiptera 12 | 0 | 0.00E+00 | 0.00E+00 | 0.00E+00 | 16 | 1.13E-02 | -4.49E+00 | -5.05E-02 | 1 | 3.85E-03 | -5.56E+00 | -2.14E-02 |
| | Hemiptera 13 | 0 | 0.00E+00 | 0.00E+00 | 0.00E+00 | 0 | 0.00E+00 | 0.00E+00 | 0.00E+00 | 1 | 3.85E-03 | -5.56E+00 | -2.14E-02 |
| | Hemiptera 14 | 1 | 8.77E-03 | -4.74E+00 | -4.15E-02 | 1 | 7.04E-04 | -7.26E+00 | -5.11E-03 | 0 | 0.00E+00 | 0.00E+00 | 0.00E+00 |
| | Hemiptera 15 | 0 | 0.00E+00 | 0.00E+00 | 0.00E+00 | 4 | 2.82E-03 | -5.87E+00 | -1.65E-02 | 0 | 0.00E+00 | 0.00E+00 | 0.00E+00 |
| | Hemiptera 16 | 1 | 8.77E-03 | -4.74E+00 | -4.15E-02 | 1 | 7.04E-04 | -7.26E+00 | -5.11E-03 | 0 | 0.00E+00 | 0.00E+00 | 0.00E+00 |
| | Hemiptera 17 | 0 | 0.00E+00 | 0.00E+00 | 0.00E+00 | 2 | 1.41E-03 | -6.57E+00 | -9.25E-03 | 0 | 0.00E+00 | 0.00E+00 | 0.00E+00 |
| | Hemiptera 18 | 0 | 0.00E+00 | 0.00E+00 | 0.00E+00 | 2 | 1.41E-03 | -6.57E+00 | -9.25E-03 | 0 | 0.00E+00 | 0.00E+00 | 0.00E+00 |
| | Hemiptera 19 | 0 | 0.00E+00 | 0.00E+00 | 0.00E+00 | 6 | 4.23E-03 | -5.47E+00 | -2.31E-02 | 0 | 0.00E+00 | 0.00E+00 | 0.00E+00 |
| | Hemiptera 20 | 0 | 0.00E+00 | 0.00E+00 | 0.00E+00 | 2 | 1.41E-03 | -6.57E+00 | -9.25E-03 | 0 | 0.00E+00 | 0.00E+00 | 0.00E+00 |
| | Hemiptera 21 | 0 | 0.00E+00 | 0.00E+00 | 0.00E+00 | 3 | 2.11E-03 | -6.16E+00 | -1.30E-02 | 0 | 0.00E+00 | 0.00E+00 | 0.00E+00 |
| | Hemiptera 22 | 0 | 0.00E+00 | 0.00E+00 | 0.00E+00 | 18 | 1.27E-02 | -4.37E+00 | -5.54E-02 | 1 | 3.85E-03 | -5.56E+00 | -2.14E-02 |
| | Hemiptera 23 | 0 | 0.00E+00 | 0.00E+00 | 0.00E+00 | 2 | 1.41E-03 | -6.57E+00 | -9.25E-03 | 0 | 0.00E+00 | 0.00E+00 | 0.00E+00 |
| | Hemiptera 24 | 0 | 0.00E+00 | 0.00E+00 | 0.00E+00 | 4 | 2.82E-03 | -5.87E+00 | -1.65E-02 | 0 | 0.00E+00 | 0.00E+00 | 0.00E+00 |
| | Hemiptera 25 | 0 | 0.00E+00 | 0.00E+00 | 0.00E+00 | 29 | 2.04E-02 | -3.89E+00 | -7.95E-02 | 1 | 3.85E-03 | -5.56E+00 | -2.14E-02 |
| | Hemiptera 26 | 0 | 0.00E+00 | 0.00E+00 | 0.00E+00 | 11 | 7.75E-03 | -4.86E+00 | -3.77E-02 | 0 | 0.00E+00 | 0.00E+00 | 0.00E+00 |
| | Hemiptera 27 | 0 | 0.00E+00 | 0.00E+00 | 0.00E+00 | 3 | 2.11E-03 | -6.16E+00 | -1.30E-02 | 0 | 0.00E+00 | 0.00E+00 | 0.00E+00 |
| | Hemiptera 28 | 0 | 0.00E+00 | 0.00E+00 | 0.00E+00 | 1 | 7.04E-04 | -7.26E+00 | -5.11E-03 | 0 | 0.00E+00 | 0.00E+00 | 0.00E+00 |
| Himenoptera | Himenoptera 1 | 0 | 0.00E+00 | 0.00E+00 | 0.00E+00 | 2 | 1.41E-03 | -6.57E+00 | -9.25E-03 | 0 | 0.00E+00 | 0.00E+00 | 0.00E+00 |
| | Himenoptera 2 | 0 | 0.00E+00 | 0.00E+00 | 0.00E+00 | 6 | 4.23E-03 | -5.47E+00 | -2.31E-02 | 0 | 0.00E+00 | 0.00E+00 | 0.00E+00 |
| | Himenoptera 3 | 0 | 0.00E+00 | 0.00E+00 | 0.00E+00 | 2 | 1.41E-03 | -6.57E+00 | -9.25E-03 | 0 | 0.00E+00 | 0.00E+00 | 0.00E+00 |
| | Himenoptera 4 | 1 | 8.77E-03 | -4.74E+00 | -4.15E-02 | 1 | 7.04E-04 | -7.26E+00 | -5.11E-03 | 2 | 7.69E-03 | -4.87E+00 | -3.74E-02 |
| | Himenoptera 5 | 0 | 0.00E+00 | 0.00E+00 | 0.00E+00 | 4 | 2.82E-03 | -5.87E+00 | -1.65E-02 | 1 | 3.85E-03 | -5.56E+00 | -2.14E-02 |
| | Himenoptera 6 | 0 | 0.00E+00 | 0.00E+00 | 0.00E+00 | 5 | 3.52E-03 | -5.65E+00 | -1.99E-02 | 0 | 0.00E+00 | 0.00E+00 | 0.00E+00 |

**Table S2.** Data used for the calculation of the Shannon-Wiener index.

| | | | | | | | | | | | | | |
|---|---|---|---|---|---|---|---|---|---|---|---|---|---|
| | Himenoptera 7 | 1 | 8.77E-03 | -4.74E+00 | -4.15E-02 | 1 | 7.04E-04 | -7.26E+00 | -5.11E-03 | 0 | 0.00E+00 | 0.00E+00 | 0.00E+00 |
| | Himenoptera 8 | 0 | 0.00E+00 | 0.00E+00 | 0.00E+00 | 2 | 1.41E-03 | -6.57E+00 | -9.25E-03 | 1 | 3.85E-03 | -5.56E+00 | -2.14E-02 |
| | Himenoptera 9 | 0 | 0.00E+00 | 0.00E+00 | 0.00E+00 | 4 | 2.82E-03 | -5.87E+00 | -1.65E-02 | 2 | 7.69E-03 | -4.87E+00 | -3.74E-02 |
| | Himenoptera 10 | 0 | 0.00E+00 | 0.00E+00 | 0.00E+00 | 4 | 2.82E-03 | -5.87E+00 | -1.65E-02 | 1 | 3.85E-03 | -5.56E+00 | -2.14E-02 |
| | Himenoptera 11 | 1 | 8.77E-03 | -4.74E+00 | -4.15E-02 | 0 | 0.00E+00 | 0.00E+00 | 0.00E+00 | 0 | 0.00E+00 | 0.00E+00 | 0.00E+00 |
| | Himenoptera 12 | 2 | 1.75E-02 | -4.04E+00 | -7.09E-02 | 1 | 7.04E-04 | -7.26E+00 | -5.11E-03 | 0 | 0.00E+00 | 0.00E+00 | 0.00E+00 |
| | Himenoptera 13 | 0 | 0.00E+00 | 0.00E+00 | 0.00E+00 | 1 | 7.04E-04 | -7.26E+00 | -5.11E-03 | 1 | 3.85E-03 | -5.56E+00 | -2.14E-02 |
| | Himenoptera 14 | 0 | 0.00E+00 | 0.00E+00 | 0.00E+00 | 1 | 7.04E-04 | -7.26E+00 | -5.11E-03 | 0 | 0.00E+00 | 0.00E+00 | 0.00E+00 |
| Lepidoptera | Lepidoptera 1 | 0 | 0.00E+00 | 0.00E+00 | 0.00E+00 | 1 | 7.04E-04 | -7.26E+00 | -5.11E-03 | 0 | 0.00E+00 | 0.00E+00 | 0.00E+00 |
| | Lepidoptera 2 | 0 | 0.00E+00 | 0.00E+00 | 0.00E+00 | 2 | 1.41E-03 | -6.57E+00 | -9.25E-03 | 0 | 0.00E+00 | 0.00E+00 | 0.00E+00 |
| | Lepidoptera 3 | 1 | 8.77E-03 | -4.74E+00 | -4.15E-02 | 11 | 7.75E-03 | -4.86E+00 | -3.77E-02 | 0 | 0.00E+00 | 0.00E+00 | 0.00E+00 |
| | Lepidoptera 4 | 1 | 8.77E-03 | -4.74E+00 | -4.15E-02 | 7 | 4.93E-03 | -5.31E+00 | -2.62E-02 | 0 | 0.00E+00 | 0.00E+00 | 0.00E+00 |
| | Lepidoptera 5 | 0 | 0.00E+00 | 0.00E+00 | 0.00E+00 | 2 | 1.41E-03 | -6.57E+00 | -9.25E-03 | 0 | 0.00E+00 | 0.00E+00 | 0.00E+00 |
| | Lepidoptera 6 | 2 | 1.75E-02 | -4.04E+00 | -7.09E-02 | 1 | 7.04E-04 | -7.26E+00 | -5.11E-03 | 0 | 0.00E+00 | 0.00E+00 | 0.00E+00 |
| | Lepidoptera 7 | 0 | 0.00E+00 | 0.00E+00 | 0.00E+00 | 2 | 1.41E-03 | -6.57E+00 | -9.25E-03 | 0 | 0.00E+00 | 0.00E+00 | 0.00E+00 |
| | Lepidoptera 8 | 0 | 0.00E+00 | 0.00E+00 | 0.00E+00 | 1 | 7.04E-04 | -7.26E+00 | -5.11E-03 | 0 | 0.00E+00 | 0.00E+00 | 0.00E+00 |
| | Lepidoptera 9 | 0 | 0.00E+00 | 0.00E+00 | 0.00E+00 | 13 | 9.15E-03 | -4.69E+00 | -4.30E-02 | 0 | 0.00E+00 | 0.00E+00 | 0.00E+00 |
| | Lepidoptera 10 | 0 | 0.00E+00 | 0.00E+00 | 0.00E+00 | 3 | 2.11E-03 | -6.16E+00 | -1.30E-02 | 0 | 0.00E+00 | 0.00E+00 | 0.00E+00 |
| | Lepidoptera 11 | 0 | 0.00E+00 | 0.00E+00 | 0.00E+00 | 1 | 7.04E-04 | -7.26E+00 | -5.11E-03 | 0 | 0.00E+00 | 0.00E+00 | 0.00E+00 |
| | Lepidoptera 12 | 0 | 0.00E+00 | 0.00E+00 | 0.00E+00 | 3 | 2.11E-03 | -6.16E+00 | -1.30E-02 | 0 | 0.00E+00 | 0.00E+00 | 0.00E+00 |
| | Lepidoptera 13 | 0 | 0.00E+00 | 0.00E+00 | 0.00E+00 | 6 | 4.23E-03 | -5.47E+00 | -2.31E-02 | 0 | 0.00E+00 | 0.00E+00 | 0.00E+00 |
| | Lepidoptera 14 | 0 | 0.00E+00 | 0.00E+00 | 0.00E+00 | 1 | 7.04E-04 | -7.26E+00 | -5.11E-03 | 0 | 0.00E+00 | 0.00E+00 | 0.00E+00 |
| | Lepidoptera 15 | 0 | 0.00E+00 | 0.00E+00 | 0.00E+00 | 1 | 7.04E-04 | -7.26E+00 | -5.11E-03 | 0 | 0.00E+00 | 0.00E+00 | 0.00E+00 |
| | Lepidoptera 16 | 0 | 0.00E+00 | 0.00E+00 | 0.00E+00 | 3 | 2.11E-03 | -6.16E+00 | -1.30E-02 | 0 | 0.00E+00 | 0.00E+00 | 0.00E+00 |
| | Lepidoptera 17 | 0 | 0.00E+00 | 0.00E+00 | 0.00E+00 | 2 | 1.41E-03 | -6.57E+00 | -9.25E-03 | 0 | 0.00E+00 | 0.00E+00 | 0.00E+00 |
| | Lepidoptera 18 | 0 | 0.00E+00 | 0.00E+00 | 0.00E+00 | 1 | 7.04E-04 | -7.26E+00 | -5.11E-03 | 0 | 0.00E+00 | 0.00E+00 | 0.00E+00 |
| | Lepidoptera 19 | 0 | 0.00E+00 | 0.00E+00 | 0.00E+00 | 2 | 1.41E-03 | -6.57E+00 | -9.25E-03 | 0 | 0.00E+00 | 0.00E+00 | 0.00E+00 |
| | Lepidoptera 20 | 0 | 0.00E+00 | 0.00E+00 | 0.00E+00 | 1 | 7.04E-04 | -7.26E+00 | -5.11E-03 | 0 | 0.00E+00 | 0.00E+00 | 0.00E+00 |

**Table S2.** Data used for the calculation of the Shannon-Wiener index.

| | | | | | | | | | | | | | |
|---|---|---|---|---|---|---|---|---|---|---|---|---|---|
| | Lepidoptera 21 | 0 | 0.00E+00 | 0.00E+00 | 0.00E+00 | 1 | 7.04E-04 | -7.26E+00 | -5.11E-03 | 0 | 0.00E+00 | 0.00E+00 | 0.00E+00 |
| | Lepidoptera 22 | 0 | 0.00E+00 | 0.00E+00 | 0.00E+00 | 3 | 2.11E-03 | -6.16E+00 | -1.30E-02 | 0 | 0.00E+00 | 0.00E+00 | 0.00E+00 |
| | Lepidoptera 23 | 0 | 0.00E+00 | 0.00E+00 | 0.00E+00 | 2 | 1.41E-03 | -6.57E+00 | -9.25E-03 | 0 | 0.00E+00 | 0.00E+00 | 0.00E+00 |
| Neuroptera | Neuroptera 1 | 0 | 0.00E+00 | 0.00E+00 | 0.00E+00 | 6 | 4.23E-03 | -5.47E+00 | -2.31E-02 | 0 | 0.00E+00 | 0.00E+00 | 0.00E+00 |
| Psocoptera | Psocoptera 1 | 1 | 8.77E-03 | -4.74E+00 | -4.15E-02 | 6 | 4.23E-03 | -5.47E+00 | -2.31E-02 | 9 | 3.46E-02 | -3.36E+00 | -1.16E-01 |
| | Psocoptera 2 | 3 | 2.63E-02 | -3.64E+00 | -9.57E-02 | 88 | 6.20E-02 | -2.78E+00 | -1.72E-01 | 92 | 3.54E-01 | -1.04E+00 | -3.68E-01 |
| | Psocoptera 3 | 1 | 8.77E-03 | -4.74E+00 | -4.15E-02 | 13 | 9.15E-03 | -4.69E+00 | -4.30E-02 | 12 | 4.62E-02 | -3.08E+00 | -1.42E-01 |
| | Psocoptera 4 | 0 | 0.00E+00 | 0.00E+00 | 0.00E+00 | 6 | 4.23E-03 | -5.47E+00 | -2.31E-02 | 3 | 1.15E-02 | -4.46E+00 | -5.15E-02 |
| | Psocoptera 5 | 2 | 1.75E-02 | -4.04E+00 | -7.09E-02 | 14 | 9.86E-03 | -4.62E+00 | -4.55E-02 | 6 | 2.31E-02 | -3.77E+00 | -8.70E-02 |
| | Psocoptera 6 | 0 | 0.00E+00 | 0.00E+00 | 0.00E+00 | 4 | 2.82E-03 | -5.87E+00 | -1.65E-02 | 5 | 1.92E-02 | -3.95E+00 | -7.60E-02 |
| | Psocoptera 7 | 0 | 0.00E+00 | 0.00E+00 | 0.00E+00 | 1 | 7.04E-04 | -7.26E+00 | -5.11E-03 | 1 | 3.85E-03 | -5.56E+00 | -2.14E-02 |
| | Psocoptera 8 | 0 | 0.00E+00 | 0.00E+00 | 0.00E+00 | 1 | 7.04E-04 | -7.26E+00 | -5.11E-03 | 0 | 0.00E+00 | 0.00E+00 | 0.00E+00 |
| Thysanoptera | Thysanoptera 1 | 0 | 0.00E+00 | 0.00E+00 | 0.00E+00 | 0 | 0.00E+00 | 0.00E+00 | 0.00E+00 | 2 | 7.69E-03 | -4.87E+00 | -3.74E-02 |
| | Thysanoptera 2 | 1 | 8.77E-03 | -4.74E+00 | -4.15E-02 | 0 | 0.00E+00 | 0.00E+00 | 0.00E+00 | 0 | 0.00E+00 | 0.00E+00 | 0.00E+00 |
| | Thysanoptera 3 | 0 | 0.00E+00 | 0.00E+00 | 0.00E+00 | 0 | 0.00E+00 | 0.00E+00 | 0.00E+00 | 1 | 3.85E-03 | -5.56E+00 | -2.14E-02 |
| Trichoptera | Trichoptera 1 | 0 | 0.00E+00 | 0.00E+00 | 0.00E+00 | 4 | 2.82E-03 | -5.87E+00 | -1.65E-02 | 0 | 0.00E+00 | 0.00E+00 | 0.00E+00 |
| | Trichoptera 2 | 0 | 0.00E+00 | 0.00E+00 | 0.00E+00 | 1 | 7.04E-04 | -7.26E+00 | -5.11E-03 | 0 | 0.00E+00 | 0.00E+00 | 0.00E+00 |
| | Trichoptera 3 | 0 | 0.00E+00 | 0.00E+00 | 0.00E+00 | 1 | 7.04E-04 | -7.26E+00 | -5.11E-03 | 0 | 0.00E+00 | 0.00E+00 | 0.00E+00 |
| Total | | 114 | | | | 1420 | | | | 260 | | | |
| Shannon-Wiener Index | | Negative control (Unilluminated) | | | **2.80** | Positive control (Metal halide) | | | **4.07** | CromaLux | | | **2.95** |
